# A novel method of carotid artery wall imaging: black-blood CT

**DOI:** 10.1007/s00330-023-10247-5

**Published:** 2023-09-22

**Authors:** Yao Lu, Ruoyao Cao, Sheng Jiao, Ling Li, Chao Liu, Hailong Hu, Zhuangfei Ma, Yun Jiang, Juan Chen

**Affiliations:** 1 The Key Laboratory of Geriatrics, Beijing Institute of GeriatricsInstitute of Geriatric Medicine, Chinese Academy of Medical Sciences, Beijing Hospital/National Center of Gerontology of National Health Commission, Beijing, People’s Republic of China; 2 Department of Radiology, Beijing Hospital, National Center of Gerontology, Institute of Geriatric Medicine, Chinese Academy of Medical Sciences, No.1, DaHua Road, Dong Dan, Beijing, 100730 People’s Republic of China; 3 Graduate School of Peking Union Medical College, Beijing, People’s Republic of China; 4 Department of Radiology, Beijing Shijitan Hospital, Capital Medical University, Beijing, People’s Republic of China; 5 CT Clinical Research Department, CT Business Unit, Canon Medical Systems (China) CO., LTD., Beijing, People’s Republic of China; 6 Department of Neurology, Beijing Hospital, National Center of Gerontology, Institute of Geriatric Medicine, Chinese Academy of Medical Sciences, Beijing, People’s Republic of China

**Keywords:** Computed tomography angiography, Carotid artery plaque, Subtraction technique, Magnetic resonance imaging

## Abstract

**Objectives:**

To evaluate the application of black-blood CT (BBCT) in carotid artery wall imaging and its accuracy in disclosing stenosis rate and plaque burden of carotid artery.

**Methods:**

A total of 110 patients underwent contrast-enhanced CT scan with two phases, and BBCT images were obtained using contrast-enhancement (CE)-boost technology. Two radiologists independently scored subjective image quality on black-blood computerized tomography (BBCT) images using a 4-point scale and then further analyzed plaque types. The artery stenosis rate on BBCT was measured and compared with CTA. The plaque burden on BBCT was compared with that on high-resolution intracranial vessel wall MR imaging (VW-MR imaging). The kappa value and intraclass correlation coefficient (ICC) were used for consistency analysis. The diagnostic accuracy of BBCT for stenosis rate and plaque burden greater than 50% was evaluated by AUC.

**Results:**

The subjective image quality scores of BBCT had good consistency between the two readers (ICC = 0.836, *p* < 0.001). BBCT and CTA had a good consistency in the identification of stenosis rate (*p* < 0.001). There was good consistency between BBCT and VW-MR in diagnosis of plaque burden (*p* < 0.001). As for plaque burden over 50%, BBCT had good sensitivity (93.10%) and specificity (73.33%), with an AUC of 0.950 (95%CI 0.838–0.993). Compared with CTA, BBCT had higher consistency with VW-MR in disclosing low-density plaques and mixed plaques (ICC = 0.931 vs 0.858, *p* < 0.001).

**Conclusions:**

BBCT can not only display the carotid artery wall clearly but also accurately diagnose the stenosis rate and plaque burden of carotid artery.

**Clinical relevance statement:**

Black-blood CT, as a novel imaging technology, can assist clinicians and radiologists in better visualizing the structure of the vessel wall and plaques, especially for patients with contraindication to MRI.

**Key Points:**

• *Black-blood CT can clearly visualize the carotid artery wall and plaque burden.*

• *Black-blood CT is superior to conventional CTA with more accurate diagnosis of the carotid stenosis rate and plaque burden features.*

## Introduction

Carotid atherosclerotic plaque, especially vulnerable plaque, is one of the important risk factors for ischemic stroke [1]. The vulnerability of carotid plaque and the stenosis extent of carotid artery are the key issues that clinicians concern [2, 3]. Various carotid artery imaging are helpful in identifying atherosclerotic plaques, such as ultrasound, conventional computed tomography angiography (CTA), magnetic resonance imaging (MRI), and digital subtraction angiography (DSA) [4]. Many clinical studies have shown that unstable plaque and stenosis of carotid artery are closely related to ipsilateral stroke [5]. VW-MR imaging is superior to CTA on revealing the arterial wall features and plaque components [6, 7], but VW-MR imaging takes a long scan time, and the claustrophobic patients and the patients with some metal implants are not suitable to MRI [8]. Although qualitative diagnostic methods for plaque have been established using ultrasound and MRI, CT is indispensable for evaluating carotid artery stenosis and plaque for the following reasons: (1) Clinically, CT and CTA are the most commonly used examination in diagnosis of cerebrovascular disease events with the advantages of fast scanning and few contraindications. Doctors can quickly obtain the images and assess the carotid artery stenosis accurately. Besides this, CTA also has the advantages of arbitrary orientation reconstruction and sensitively displaying calcification. (2) Compared with ultrasound, CT is more objective and less affected by the operator. Thus, CT is still kept in the first line of the daily clinical work. However, CTA has limitations in displaying artery wall and vulnerable plaque components.

**Fig. 1:**
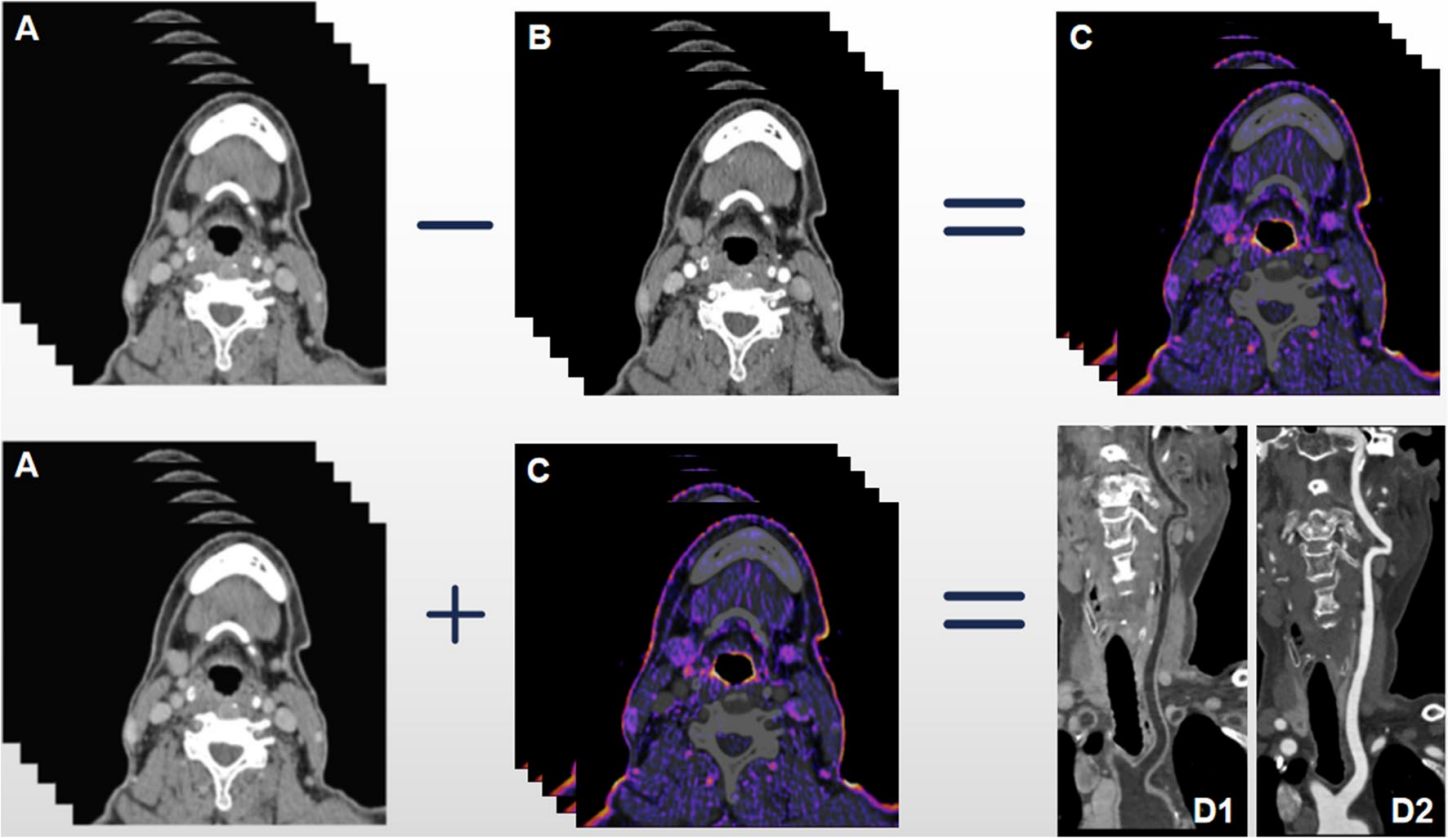
The principle of BBCT technique. Iodine mappings (**C**) were obtained by subtracting arterial-phase CT images (**B**) from the delayed-phase images (**A**). BBCT (D1) were obtained by adding delayed-phase images to iodine mappings, which displayed artery wall clearly. Conventional CTA (D2) poorly displayed with this WL/WW settings

Contrast-enhancement-boost (CE-boost) is one of the CT technologies using subtraction technique and registration algorithm, which attenuates the motion influence of different phases and provides accurate registration [9]. CE-boost can enhance the brightness of blood vessels by subtracting non-contrast CT images from enhanced-contrast CT images [10]. Similar with CE-boost, subtraction technology and registration algorithm are also used in generating BBCT. A set of iodine mappings are acquired by subtracting arterial-phase CT images from the delayed-phase images, and then, the BBCT images are obtained by superimposing the iodine images on the delayed-phase CT images. Thus, the vascular lumen turns to be black on both BBCT images and the iodine mappings. A previous study has shown that delayed-phase CT images can provide more information of plaques [11]. As the vascular wall has the characteristics of delayed enhancement, the lumen and wall structure of carotid artery can be distinguished on BBCT by superimposing the delayed-phase images and iodine mappings. BBCT improves the exhibition of the arterial wall, which is helpful in the evaluation of carotid stenosis and plaque burden (Fig. 1).

It is well known that unstable carotid plaque and stenosis are closely related to ischemic stroke; in this study, we presented a novel method of carotid artery wall imaging using BBCT to evaluate not only stenosis but also the plaque burden of the carotid arteries. Meanwhile, the image quality and diagnostic value of BBCT in carotid artery imaging were evaluated. The stenosis degree and plaque features on BBCT were analyzed and compared with those on conventional CTA and VW-MR imaging.

## Methods

### Study population

From January to May 2022, a total of 110 patients received carotid artery enhanced CT scans in our hospital, including 85 males (77.3%) and 25 females (22.7%), with the mean age of 68.7 ± 9.1 years. Inclusion criteria were as follows: (1) age ≥ 18 years old; (2) atherosclerotic plaques in the cervical carotid artery confirmed by ultrasound, CT, or MRI; (3) less than 2 weeks’ interval between VW-MRI and BBCT. Exclusion criteria were as follows: (1) history of iodine allergy; (2) patients with severe heart, liver, lung, renal failure, or hematological diseases; (3) severe artifact. Six patients were excluded due to severe image artifacts. The reason was that the patients were seriously weak and dysphoria during the examination. This study was approved by the local Institutional Research Ethics Committee, and informed consent was obtained from all patients.

### CT scan techniques and image postprocessing

All CT angiography examinations were performed on a 320-row CT scanner (Aquilion ONE Genesis, Canon Medical System). Nonionic iodine contrast agent (370 mg/ml) was intravenously injected with the dosage of 0.6 ml/kg, followed by 30 ml saline with a two-channel high-pressure injector. After contrast injection, two phases scanning of CT angiography was performed, including the arterial phase and delayed phase. Arterial-phase scanning parameters: the monitoring layer was located at the middle level of common carotid artery with manually triggered; tube voltage 120 kV, automatic milliampere. The delayed-phase scanning parameters: 150 s after injection of contrast agent, automatic trigger scanning, tube voltage of 120 kV, automatic milliampere. The scanning range of both phases was the same, from the aortic arch to the sellar with slice thickness of 1 mm.

The BBCT images were obtained by subtracting the arterial-phase images from the delayed-phase images using the analysis software installed in the CT workstation (Aquilion ONE, Canon Medical Systems).

### VW-MR scan techniques

All scans were performed using a 3.0-T MR scanner (Magnetom Prisma, Siemens Healthineers) with 64-channel coil. The imaging sequences included T1-weighted imaging (TIWI) and contrast-enhanced T1-weighted imaging (CE-T1WI). TIWI using sampling perfection with application-optimized contrasts by using different flip angle evolutions (SPACE) sequence: TR 800 ms, TE 16 ms, FOV 250 mm × 164 mm, voxel size 0.6 mm × 0.6 mm × 0.6 mm. Gadopentetate dimeglumine (Magnevist, Schering) was administered intravenously (0.1 mmol/kg body weight), and T1W imaging was repeated 5 min after contrast administration.

### Subjective image quality evaluation

**Fig. 2:**
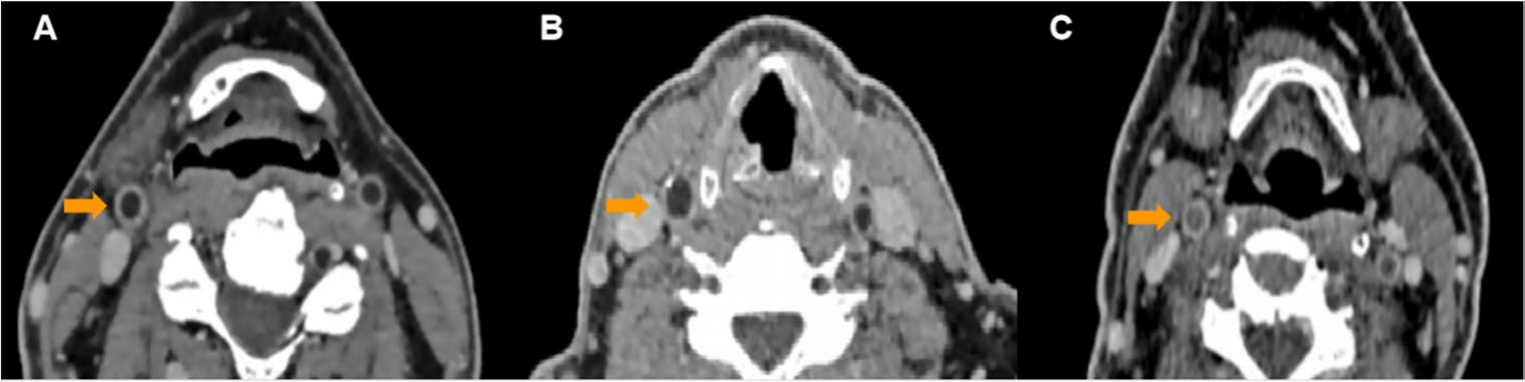
Subjective image quality evaluation of BBCT. **A** Images with a 4-point image quality score. **B** Images with a 3-point image quality score. **C** Images with a 2-point image quality score

Two radiologists independently evaluated the images of 110 patients. Readers were blinded to the patient’s clinical information. The scoring criteria were as follows: a score of 4 (best image quality) indicated high signal noise ratio (SNR), and the lumen, the wall, and the plaques were most clearly displayed; 3 (good image quality) indicated relatively high SNR, and the lumen, the wall, and the plaque were displayed well; 2 (medium image quality) indicated moderate SNR, and the lumen and wall could be easily distinguished, but the plaque was not clear enough; 1 (poor image quality) indicated low SNR, and the lumen and wall were poorly displayed, and the plaque was blurred. Points ranged from 2 to 4 indicated diagnostic quality, and points of 1 indicated nondiagnostic quality (Fig. 2).

### Comparison between BBCT and CTA

#### Carotid artery plaque recognition

Two radiologists independently analyzed carotid artery plaque on BBCT images using commercial three-dimensional (3D) workstation software (RadiAnt DICOM Viewer, Medixant). Two hundred and twenty vessels from 110 patients were analyzed. The plaque at the narrowest level of the carotid artery was selected for analysis. Calcified plaque, mixed plaque, low-density plaque, and no plaque were recorded as categorical variables 1 to 4, respectively. The calcified plaque was defined with a CT value greater than 130HU, low-density plaque was made of fat and fibers, some with intra-plaque hemorrhage, and mixed plaque contained both calcified and low-density plaque. BBCT results of reader 1 and reader 2 are denoted as BBCT_R1_ and BBCT_R2_, respectively. CTA was reviewed by a senior radiologist. The plaque types on BBCT were compared with those on conventional CTA.

#### Degree of vascular stenosis

**Fig. 3:**
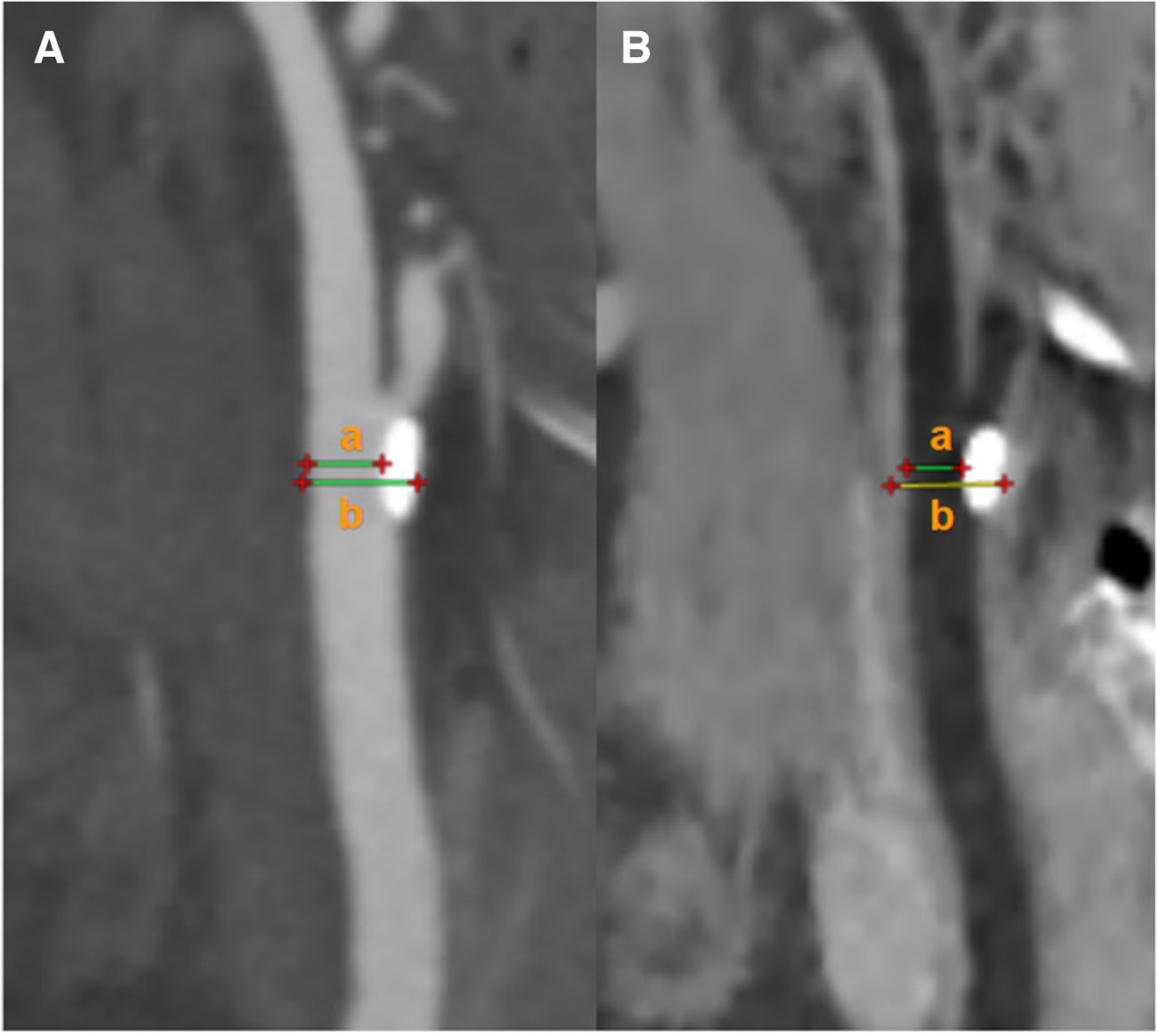
Measurement of stenosis rate. **A** CPR image of CTA. **B** CPR image of BBCT. There was a calcified plaque in the vessel wall of the common carotid artery. Stenosis rate = (1 − *a*/*b*)*100%

Carotid artery stenosis rates were measured according to the European Carotid Surgery Trial (ECST) [12]. Carotid arteries with plaque diameter larger than 5 mm were selected for stenosis rate calculation. All CTA and BBCT original images were loaded in the workstation first to generate curved planar reconstruction (CPR) images. Then, two experienced radiologists checked images and made a manual correction to ensure the centerline was in the center of the lumen. The stenosis rate was measured on CPR images where the plaques were shown best, and the measurement positions were located at the narrowest level of the lumen doublechecked on three consecutive CPR images. The mean value of the measurement results on the three consecutive CPR images was used (Fig. 3).

### Comparison of plaque burden between BBCT and VW-MR imaging

Twenty-two of 110 patients underwent VW-MR imaging. The carotid vascular area and lumen area at the most stenotic level were manually delineated on both BBCT images and VW-MR enhanced images; it takes about 3–5 min to sketch each plaque burden. Alignment was performed using software Radiant DICOM viewer with carotid bifurcation or bone structure as the reference. Sagittal or coronal reconstruction will be used if necessary. The vascular area included the vascular wall area and lumen area. The plaque burden was calculated using the following formula [13, 14]: $\mathrm{plaque}\;\mathrm{burden}\;\left(\%\right)=\left[\left(\mathrm{vascular}\;\mathrm{area}-\mathrm{lumen}\;\mathrm{area}\right)/\mathrm{vascular}\;\mathrm{area}\right]\times100\%$.

**Fig. 4:**
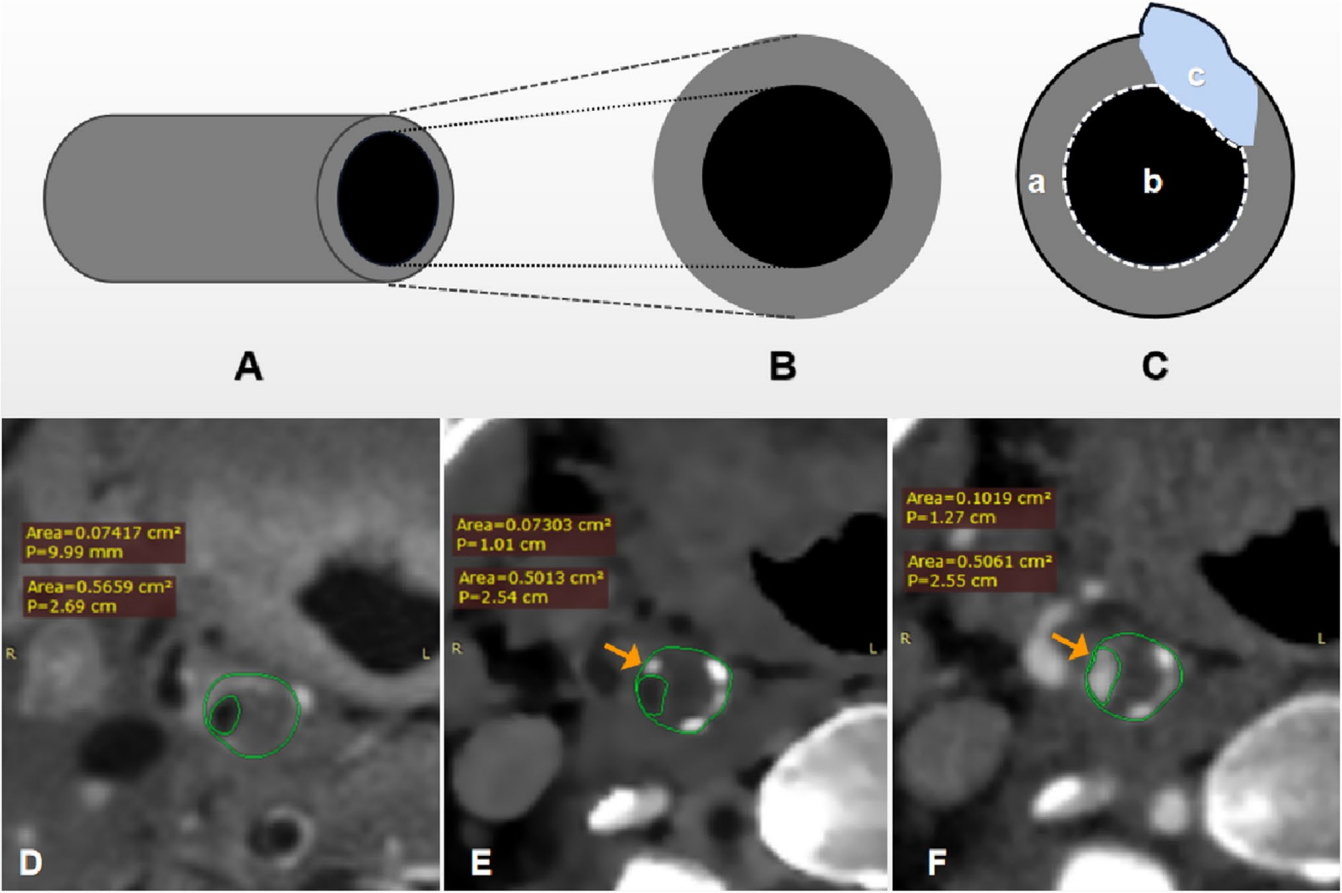
Measurement of plaque burden. Panels **B** and **C** were the cross sections of the vessel (**A**). Panel **C** was the measurement method of vessel area and lumen area. All areas in the solid black line were vascular areas (a + b + c), and the vessel wall was a, the lumen area was in the dashed white line (b), and the plaque was c. **D**, **E** Measurements of VW-MRI, BBCT, and CTA. A punctate calcified plaque (arrow) on CTA image (**F**) was similar to the lumen density resulting in measurement error. BBCT (**E**) had advantages in avoiding this error

All the low-density plaques and mixed plaques were identified in 44 vessels. The plaque burden was analyzed at the level where these plaques were disclosed on BBCT, CTA, and VW-MR images. Then two radiologists measured and compared the plaque volume on BBCT and VW-MRI using software ITK-SNAP 3.6 (Fig. 4).

### Statistical analysis

IBM SPSS 26.0 and MedCalc 19.3 software were used for statistical analysis. Numerical variables were expressed as mean ± standard deviation (SD). Two-tailed *p* < 0.05 indicated a significant difference. The consistency of diagnostic accuracy was evaluated by *κ* statistics. The *κ* value indicates the degree of consistency: 0–0.20, slight agreement; 0.21–0.40, fair agreement; 0.41–0.60, moderate agreement; 0.61–0.80, substantial agreement; and 0.81–1.00, perfect agreement. The consistency of the inter-observer image quality score, the stenosis rates of BBCT and CTA, and the plaque burden of BBCT, CTA, and VW-MR imaging were evaluated by intraclass correlation coefficient (ICC). Receiver operating characteristic (ROC) curve and area under curve (AUC) value were used to evaluate the diagnostic accuracy of BBCT for stenosis rate and plaque burden greater than 50%.

## Results

### Subjective image quality evaluation

Good image quality scores of BBCT were obtained from all the 110 patients; the scores of BBCT obtained by the two readers were 3.32 ± 0.67 and 3.29 ± 0.69, respectively. The consistency of image quality evaluation between the two readers was good (ICC = 0.836, *p* < 0.001). Regarding the plaque types in 220 carotid arteries, there was a high consistency between the two readers (*κ* = 0.886, *p* < 0.001). Meanwhile, the consistency values between BBCT and CTA of the two readers were moderate and high, respectively (*κ*_1_ = 0.778, *p* < 0.001; *κ*_2_ = 0.851, *p* < 0.001) (Table 1).

**Table 1:** Consistency of BBCT comparison with CTA and VW-MR

		$\overline{x }$±*s*/*n*	Value	*p*
Image score	Reader 1	3.32 ± 0.67	ICC = 0.836	< 0.001
	Reader 2	3.29 ± 0.69		
Plaque types	BBCT_R1_ and BBCT_R2_	220	*κ*_1_ = 0.886	< 0.001
	BBCT_R1_ and CTA		*κ*_2_ = 0.778	< 0.001
	BBCT_R2_ and CTA		*κ*_3_ = 0.851	< 0.001
Stenosis rate	BBCT and CTA	87	ICC = 0.859	< 0.001
			AUC = 0.933	< 0.001
Plaque burden	BBCT and VW-MR	44	ICC = 0.921	< 0.001
			AUC = 0.950	< 0.001
Low-density plaques	BBCT and VW-MR	29	ICC_1_ = 0.931	< 0.001
	CTA and VW-MR		ICC_2_ = 0.858	< 0.001
Plaque volume	BBCT_R1_ and VW-MR	29	ICC_1_ = 0.872	< 0.001
	BBCT_R2_ and VW-MR		ICC_2_ = 0.902	< 0.001

*BBCT*, black-blood CT; *VW-MR*, vessel wall magnetic resonance imaging; *CTA*, computed tomography angiography; *BBCT*_*R1*_, plaque types result of BBCT by reader 1; *BBCT*_*R2*_, plaque types result of BBCT by reader 2

### Degree of vascular stenosis

**Fig. 5:**
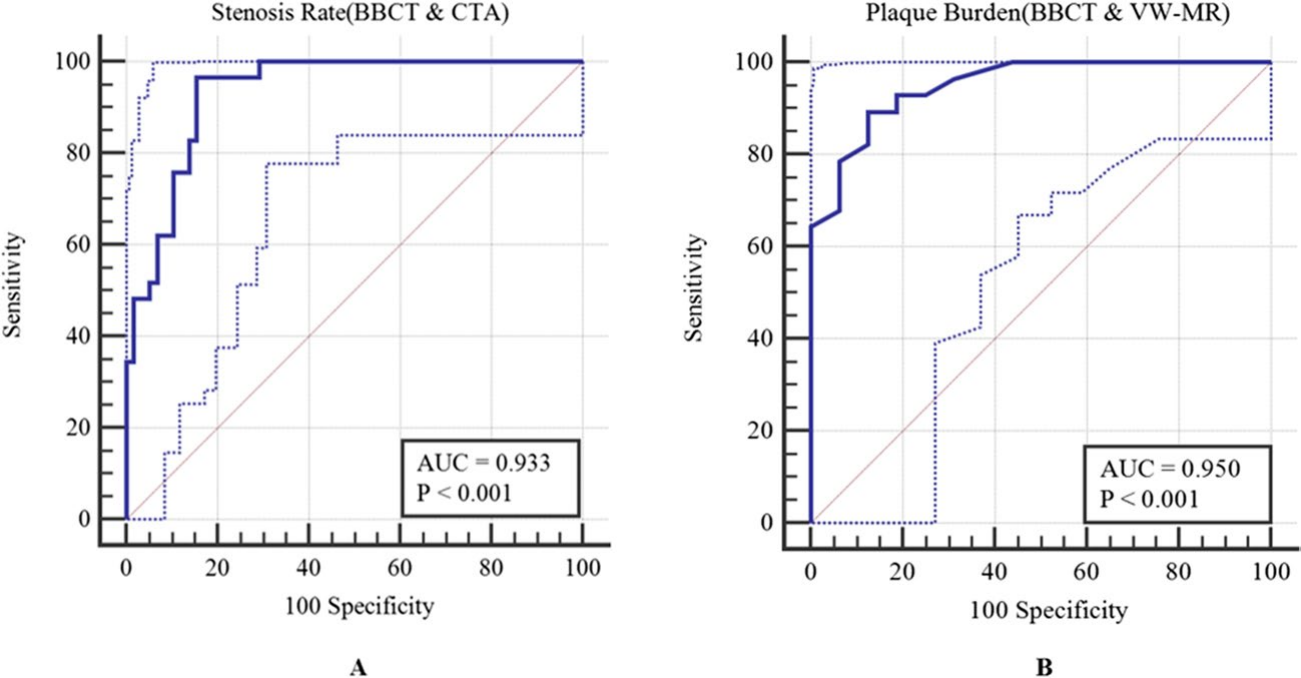
**A** Using CTA as the standard, the ROC curve of BBCT for the diagnostic accuracy of stenosis rate that greater than 50%. **B** Using VW-MR imaging as the standard, the ROC curve of BBCT for the diagnostic accuracy of plaque burden that greater than 50%

Among the 220 arteries, 87 carotid arteries were selected for stenosis rate calculation. There was a high consistency between BBCT and conventional CTA (ICC = 0.859; *p* < 0.001) in displaying the vessel stenosis. Among 87 vessels, stenosis greater than 50% was revealed in 37 vessels on BBCT, and 29 on conventional CTA (Table 2). Sensitivity and specificity of BBCT were 96.55% (28/29) and 84.48% (49/58), respectively. The AUC of BBCT for the detection of stenosis rates greater than 50% was 0.933 (95%CI 0.859–0.976, *p* < 0.001) (Fig. 5).

**Table 2:** Detection of carotid artery stenosis rate and plaque burden over 50%

		Stenosis rate (CTA)			Plaque burden (VW-MR)		
		≥ 50%	< 50%	Sum	≥ 50%	< 50%	Sum
BBCT	≥ 50%	28	9	37	27	4	31
	< 50%	1	49	50	2	11	13
Total		29	58	87	29	15	44

*BBCT*, black-blood CT; *VW-MR*, vessel wall magnetic resonance imaging; *CTA*, computed tomography angiography

### Plaque burden

Plaque burden was analyzed in 44 vessels of 22 patients who underwent VW-MR. The plaque burden was highly consistent between BBCT and VW-MR imaging (ICC = 0.921; *p* < 0.001). Plaque burden was identified greater than 50% in 31 vessels on BBCT, and in 29 vessels on VW-MR imaging (Table 2). Sensitivity and specificity of BBCT were 93.10% (27/29) and 73.33% (11/15), respectively. The AUC of BBCT for the detection of plaque burden greater than 50% was 0.950 (95%CI 0.838–0.993, *p* < 0.001) (Fig. 5).

**Fig. 6:**
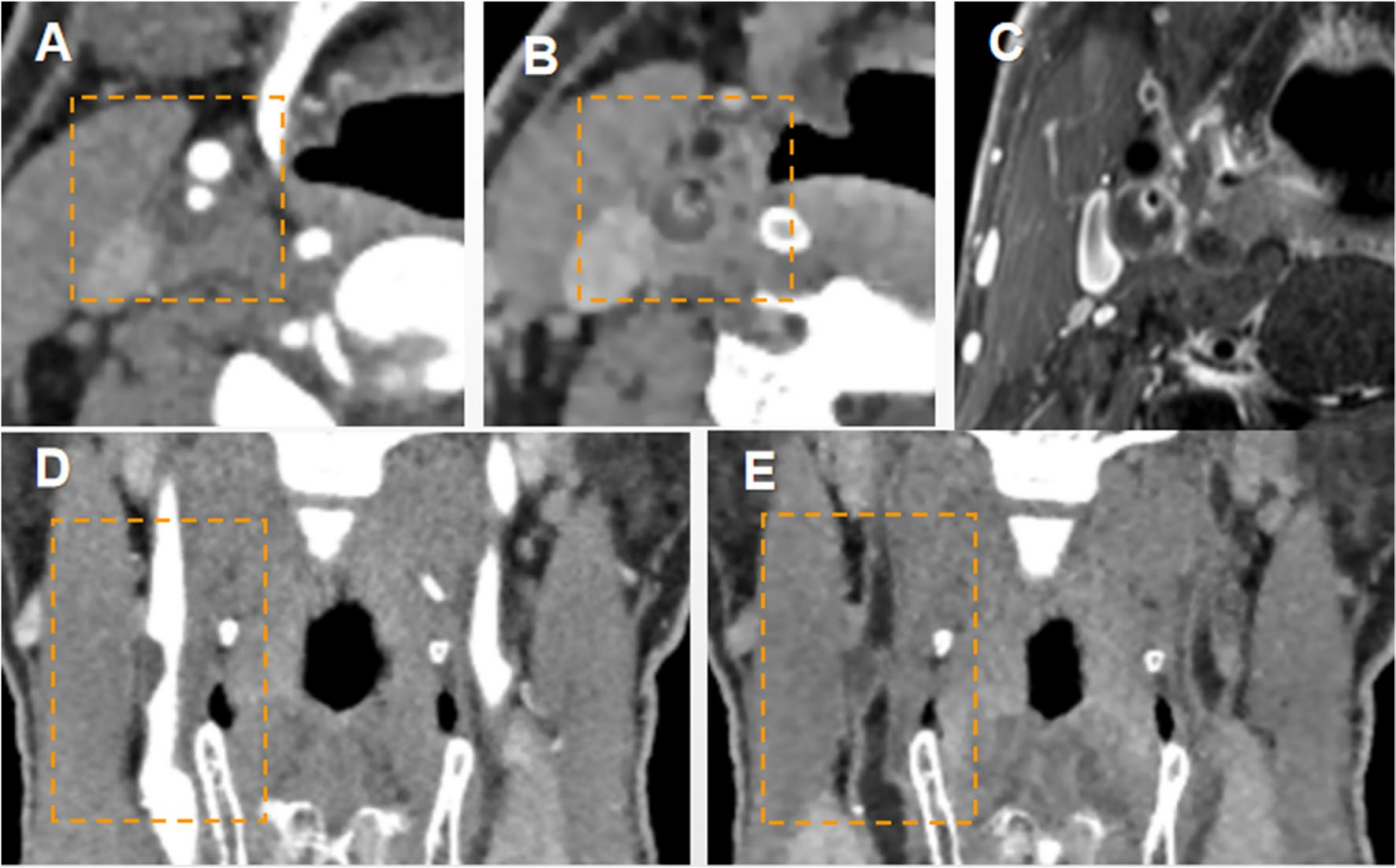
A 65-year-old man with a hypodense plaque in the right internal carotid artery which induced severe luminal stenosis. On the axial images, BBCT (**B**) was better than CTA (**A**) in displaying low-density plaques. VW-MR images (**C**) showed an eccentric vulnerable plaque; **D**, **E** Multi-planar reformatting (MPR) coronal view of CTA and BBCT, respectively

A total of 29 low-density plaques and mixed plaques were identified and measured. Compared with CTA, BBCT had higher consistency with VW-MR in disclosing low-density plaques and mixed plaques (ICC = 0.931 vs 0.858, *p* < 0.001) (Fig. 6). The plaque volume assessed by two radiologists showed a high level of consistency between BBCT and VW-MRI (ICC_1_ = 0.872, *p* < 0.001, ICC_2_ = 0.902, *p* < 0.001) (Table 1).

### Radiation dose

In this study, the radiation dose in the delayed phase was 1.84 ± 0.47 mSv, and the radiation dose of the whole scan was 3.82 ± 0.95 mSv.

## Discussion

In this study, we evaluated the subjective image quality of BBCT and its diagnostic accuracy of plaque types. The stenosis rate and plaque burden on BBCT were compared with those on conventional CTA and VW-MR imaging. The BBCT was highly consistent with CTA and VW-MR imaging in displaying carotid artery wall as well as plaque type identification. High specificity and sensitivity of carotid artery stenosis rate and plaque burden greater than 50% were obtained.

Subtraction technology has been widely used in CT [15, 16]. However, highlighting the vascular wall imaging on CT scan is still very challenging to date. In addition to carotid atherosclerosis, there are many other diseases that require imaging of the arterial wall [17–19], such as Takayasu arteritis, arterial dissection, and mural thrombus. BBCT depresses the enhanced CT value in the lumen and simultaneously highlights the presentation of the carotid artery wall. To our knowledge, this is the first study to explore the carotid artery wall features based on BBCT images utilizing the subtraction technique. Dr. Steven [20] used Cinematic rendering (CR) to generate black-blood cinematic rendering (BBCR) images for the visualization of cardiac structures. Dr. Rotzinger [21] developed a dual-energy CT algorithm and used two-material decomposition analysis to generate dark-blood CT, aiming to better visualize the aortic wall and aortic intramural hematoma. In this study, we explored BBCT on the carotid arteries. Our results showed that the BBCT can display not only the lumen stenosis but also the wall structure of the carotid artery very well. The BBCT images obtained by subtraction technology can be processed on the workstation with about 3 min on each set of images, which is easy and convenient.

CTA is well known as an effective technique for the diagnosis of carotid artery stenosis with high sensitivity and has a high consistency with DSA which is gold standard [22]. The degree of carotid artery stenosis and plaque type are the main concerns of clinicians. Thus, CTA was used as a criterion for evaluating carotid stenosis in this study, and the results of this study showed a good consistency between BBCT and CTA.

Plaque burden is one of the indicators of the severity of carotid atherosclerosis. Morphology and stability of the plaque are closely related to a previous history of cerebrovascular diseases [23]. Accurate evaluation of plaque burden is important due to its predictive value on ischemic events [24]. Ultrasound and MRI are generally used in revealing the wall and lumen of carotid artery. VW-MR has shown great value in identifying atherosclerotic plaque components compared with pathology, such as intra-plaque hemorrhage, fibrous cap disruption, and lipid-rich necrotic core [25]. We therefore used VW-MR as the gold standard for plaque burden analysis. The results of this study showed that BBCT identified plaque types and stenosis rate with high quality. Compared with CTA, the measurement of plaque burden by BBCT was more consistent with VW-MR. BBCT clearly displayed the carotid artery wall, had better diagnostic value for carotid plaque stenosis rate, and accurately identified the types of plaque. BBCT was superior to VW-MR in short scanning time and less contraindications. Moreover, MPR, curved planar reconstruction (CPR), and other postprocessing techniques can be used in BBCT to display the plaque manifestations more clearly and conveniently. Despite this, MRI still has irreplaceable advantages in the diagnosis of plaque properties.

We added a delayed phase to the examination process, and the radiation dose was 1.84 ± 0.47 mSv for the delayed phase only and the radiation dose of the whole scan was 3.82 ± 0.95 mSv. According to previous studies, dose of routine chest CT scan was about 3–7 mSv [26, 27]. Therefore, BBCT examination does not increase extra radiation risk of patients, and patients with carotid atherosclerosis benefit more from the evaluation.

This study has some limitations. First, as this is a single-center study, the number of patients who underwent VW-MR was small. Second, the available data were not sufficient for in-depth analysis on the details of low-density plaques, and we will continue the work in the future.

In conclusion, BBCT can display the carotid artery wall and lumen more precisely when compared with conventional CTA, improving the detection of low-density plaque. The combination of BBCT and CTA can further identify plaques more comprehensively. Moreover, BBCT is superior to VW-MR with less contraindication and shorter scanning time. It provides a novel option for clinicians to accurately evaluate carotid atherosclerotic plaques.

## Abbreviations

AUC: The area under the receiver operating characteristic curve
BBCT: Black-blood computerized tomography
CE-boost: Contrast-enhancement-boost
CPR: Curved planar reconstruction
CTA: Computed tomography angiography
DSA: Digital subtraction angiography
ECST: European Carotid Surgery Trial
ICC: Intraclass correlation coefficient
MPR: Multi-planar reformatting
MRI: Magnetic resonance imaging
ROC: Receiver operating characteristic
SD: Standard deviation
SNR: Signal noise ratio
VW-MR: Vessel wall magnetic resonance imaging

## Declarations

### Guarantor

The scientific guarantor of this publication is Juan Chen.

### Statistics and biometry

No complex statistical methods were necessary for this paper.

### Methodology

• Prospective

• Diagnostic or prognostic study

• Performed at one institution
